# A collection of annotated and harmonized human breast cancer transcriptome datasets, including immunologic classification

**DOI:** 10.12688/f1000research.10960.2

**Published:** 2018-02-09

**Authors:** Jessica Roelands, Julie Decock, Sabri Boughorbel, Darawan Rinchai, Cristina Maccalli, Michele Ceccarelli, Michael Black, Cris Print, Jeff Chou, Scott Presnell, Charlie Quinn, Puthen Jithesh, Najeeb Syed, Salha B.J. Al Bader, Shahinaz Bedri, Ena Wang, Francesco M. Marincola, Damien Chaussabel, Peter Kuppen, Lance D. Miller, Davide Bedognetti, Wouter Hendrickx

**Affiliations:** 1 Tumor Biology, Immunology and Therapy section, Sidra Medical and Research Center, Doha, Qatar; 2 Qatar Biomedical Research Institute, Hamad Bin Khalifa University, Qatar Foundation, Doha, Qatar; 3 Systems Biology Department, Sidra Medical and Research Center, Doha, Qatar; 4 Qatar Computing Research Institute, Doha, Qatar; 5 Department of Biochemistry, Otago School of Medical Sciences, University of Otago, Dunedin, 9054, New Zealand; 6 Department of Molecular Medicine and Pathology and Maurice Wilkins Institute, Faculty of Medical and Health Sciences, The University of Auckland, Auckland, 1142, New Zealand; 7 Department of Cancer Biology, Wake Forest School of Medicine, Winston-Salem, NC, 27157, USA; 8 Benaroya Research Institute at Virginia Mason, Seattle, WA, 98101, USA; 9 Translational Bioinformatics, Division of Biomedical Informatics Research, Sidra Medical and Research Center, Doha, Qatar; 10 Technical Bioinformatics team, Biomedical Informatics Division, Sidra Medical and Research Center, Doha, Qatar; 11 National Center for Cancer Care and Research (NCCCR), Hamad General Hospital, Doha, Qatar; 12 Weill Cornell Medicine - Qatar, Doha, Qatar; 13 Division of Translational Medicine, Research Branch, Sidra Medical and Research Center, Doha, Qatar; 14 Office of the Chief Research Officer (CRO), Research Branch, Sidra Medical and Research Center, Doha, Qatar; 15 Department of Surgery, Leiden University Medical Center, Leiden, 2333 ZA, Netherlands

**Keywords:** Breast Cancer, Immune Subtypes, Cancer Immune Phenotype, Gene Expression Browser, Immunologic Constant of Rejection

## Abstract

The increased application of high-throughput approaches in translational research has expanded the number of publicly available data repositories. Gathering additional valuable information contained in the datasets represents a crucial opportunity in the biomedical field. To facilitate and stimulate utilization of these datasets, we have recently developed an interactive data browsing and visualization web application, the Gene Expression Browser (GXB). In this note, we describe a curated compendium of 13 public datasets on human breast cancer, representing a total of 2142 transcriptome profiles. We classified the samples according to different immune based classification systems and integrated this information into the datasets. Annotated and harmonized datasets were uploaded to GXB. Study samples were categorized in different groups based on their immunologic tumor response profiles, intrinsic molecular subtypes and multiple clinical parameters. Ranked gene lists were generated based on relevant group comparisons. In this data note, we demonstrate the utility of GXB to evaluate the expression of a gene of interest, find differential gene expression between groups and investigate potential associations between variables with a specific focus on immunologic classification in breast cancer. This interactive resource is publicly available online at: http://breastcancer.gxbsidra.org/dm3/geneBrowser/list.

## Introduction

Technological progress in the field of biomedical research has resulted in an increased utilization of platforms generating information on a system-scale, e.g. genome, transcriptome and proteome. As researchers are typically willing and often required to share their data collections, the availability of ‘big data’ is expanding rapidly. At this moment, the NCBI Gene Expression Omnibus (GEO), a public repository of transcriptome profiles, holds over 2 million individual transcriptome profiles from more than 76,000 studies ( “Home - GEO - NCBI”, 2016). This large amount of available transcriptomic data provides major opportunities as well as challenges to researchers. Identification of differential gene expression in healthy versus diseased individuals, for example, has the potential to increase our understanding of the disease process, can lead to the identification of novel disease biomarkers or to the recognition of potential therapeutic targets. However, utilization of the available system-scale information can be challenging, since data repositories often lack the analytical and visualization tools needed for data assessment and interpretation. For this reason, proper analysis relies on elevated bioinformatics skills.

To overcome the challenges faced when analyzing transcriptomic data, we previously developed a web application called gene expression browser (GXB), which makes datasets more accessible and interactive ( Speake *et al.*, 2015). The application graphically visualizes gene expression data in bar chart or box plot representation and is capable of dynamically changing its interface views upon user input. GXB allows users to upload microarray data, add data annotations, which enables overlay of clinical data, explore gene rank lists based on their differential expression patterns between groups, view the data on a gene-by-gene basis and compare different datasets and diseases. These capabilities stimulate the acquisition of new knowledge from public datasets, as demonstrated by the first paper that employed GXB to identify a previously unknown role of a specific transcript during immune-mediated processes ( Rinchai *et al.*, 2015).

In recent years, a large number of transcriptional studies have been conducted with the aim to characterize breast cancer on a genetic basis. GEO holds about 1297 datasets relating to breast cancer. One of the main impacts gene expression profiling has had on our understanding of breast cancer has been through the classification of breast cancer into intrinsic molecular subtypes (IMS). Three main methods have been described to achieve this, which have the same subtypes, but actually use different gene sets to stratify the patients ( Hu *et al.*, 2006; Parker *et al.*, 2009; Sorlie *et al.*, 2003). Four major IMS of breast cancer have been identified: Luminal A, Luminal B, HER2-enriched and Basal-like. A less common molecular subtype called Claudin-low has been characterized at a later time point ( Prat *et al.*, 2010). Stratified IMS groups present critical differences in incidence, survival and response to treatment, and most importantly add prognostic information that is not provided by classical stratifications, like estrogen receptor status, histologic grade, tumor size, and node status ( Parker *et al.*, 2009).

**Figure 1.**
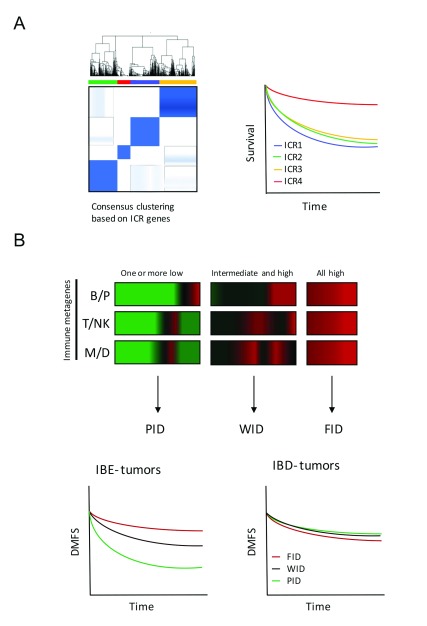
Basis of ICR and IDS/IBS classifications and prognostic value. ( **A**) Consensus clustering based on ICR genes segregates breast cancer patient in four different groups: ICR1, 2, 3 and 4. Patients with tumors categorized as ICR4 have the highest expression of the ICR gene signature and have a better prognosis compared with other ICR groups. ( **B**) Immune metagene model based on the relative expression of immune metagenes (B/P, T/NK and M/D) distinguishes PID, WID and FID tumors (horizontal axis: genes, vertical axis: individual cases). This classification has prognostic value in IBE tumors, and not in IBD tumors. Diagrams are based on Hendrickx *et al.*, 2017 ( **A**) and Miller *et al.*, 2016 ( **B**). This figure is for explanatory purposes only and does not serve as a demonstration of the GXB web application. ICR, Immunologic Constant of Rejection; IBE/D, Immune Benefit Enabled OR Disabled; F/P/WID, Favorable OR Poor OR Weak Immune Disposition.

Recent breakthroughs in the field of cancer immunotherapy and especially the application of checkpoint blockade inhibitors has ignited a fierce drive to understand the genetic basis for the huge differences observed between patients with different immune phenotypes. Several papers have shown that expression profiles are able to distinguish between those patients that have an active immune environment and those that do not ( Galon *et al.*, 2013; Herbst *et al.*, 2014; Ji *et al.*, 2012; Ribas *et al.*, 2015; Wang *et al.*, 2013). A clear correlation can be seen both regarding prognosis (survival) and prediction of therapeutic effectiveness of immune regulatory therapies. The expression of genes observed in association with tissue-specific destruction in a broader context, defined as the immunological constant of rejection (ICR), can distinguish between breast cancer patients with different prognosis. This immunological classification is based on the consensus clustering of ICR genes ( Galon *et al.*, 2013), e.g. genes underlying Th1 polarization, related chemokines, adhesion molecules and cytotoxic factors, in combination with immune regulatory genes IDO1 and FOXP3, PDCD1, CTLA4 and CD274/PD-L1 ( Figure 1A) ( Bedognetti *et al.*, 2015). In Miller *et al.* (2016), a novel survival-based immune classification system was devised for breast cancer based on the relative expression of immune gene signatures that reflect different effector immune cell subpopulations, namely antibody-producing plasma B cells (the B/P metagene), cytotoxic T and/or NK cells (the T/NK metagene), and antigen-presenting myeloid/dendritic cells (the M/D metagene). The system defines a tumor’s immune subclass based on its survival-associated immunogenic disposition status (IDS), which discriminates between *poor immunogenic disposition* (PID), *weak immunogenic disposition* (WID) and *favorable immunogenic disposition* (FID). The ability of IDS to distinguish patients with differential prognosis is dependent on the tumor’s immune benefit status (IBS), which is defined by IMS and the expression of cell proliferation markers. The IBS classification segregates immune benefit-enabled (IBE) and immune benefit-disabled (IBD) tumors. In IBE tumors, but not IBD tumors, FID status confers a protective survival benefit compared to WID and PID status ( Figure 1B) ( Miller *et al.*, 2016; Nagalla *et al.*, 2013). In this data note, we demonstrate the use of GXB to evaluate cancer gene expression across immunologic classifications of breast cancer.

Since the amount of possible datasets to be included in GXB is enormous, we chose to start with the GEO datasets underlying the immunologically classified breast cancer datasets by ( Miller *et al.*, 2016). In Hendrickx *et al.* (2017), these same datasets were classified according to ICR. This will allow us to share our immune related classifications in a comprehensible way and allow others to reuse them. A harmonization effort of the other available clinical data had been undertaken and should help the downstream analysis of the expression data. Therefore, gathering these datasets with their detailed study and sample information will facilitate the identification of clinically-relevant genetic signatures for biomarker and/or therapeutic purposes.

In this data note, using GXB, we have made available a curated compendium of 13 public datasets relevant to human breast cancer, representing a total of 2142 cases.

## Methods

### Selection of breast cancer datasets

**Figure 2.**
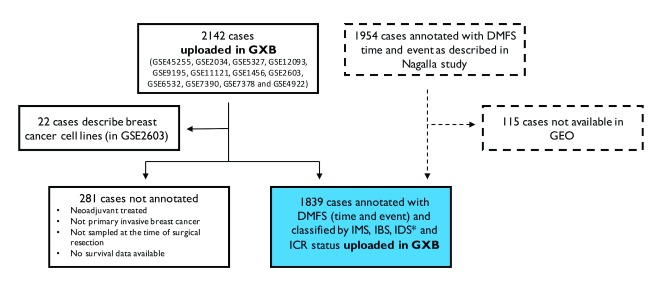
Schematic representation of dataset selection and annotation. Breast cancer cases included in 13 NCBI GEO datasets were uploaded in GXB (n=2142). 22 cases described data from breast cell lines and were excluded from our data collection. We annotated 1839 cases with survival data, IMS, IBS, IDS and ICR status. 281 cases were either neoadjuvant treated, did not represent a primary invasive tumor, were not sampled at the time of surgery or without available survival data and were therefore not annotated. The total collection includes 1839 cases from the original cohort described in Nagalla *et al.* (2013) (n=1954). Of note, 115 cases of this cohort are not included in our collection as these were not shared via GEO. *251/1839 cases have been classified for IMS “Normal-like”. IDS is not applicable for normal-like breast cancer tissue; therefore, IDS is non-classified for these samples. DMFS, Distant Metastasis Free Survival; GXB, Gene Expression Browser; IMS, intrinsic molecular subtype; IBS, immune benefit status; IDS, immune disposition status; ICR, immunologic constant of rejection.

The starting point of our selection of breast cancer datasets are the patient cohorts included in the multi-study breast cancer database described by Nagalla *et al.* (2013). These 13 NCBI GEO datasets (GEO accession numbers: GSE45255, GSE2034, GSE5327, GSE12093, GSE9195, GSE11121, GSE1456, GSE2603, GSE6532, GSE7390, GSE7378 and GSE4922) resulted in 2142 cases initially uploaded in GXB. 22 of these cases reflect data from breast cancer cell lines and were therefore excluded from our data collection. A total of 1839 cases represent primary invasive breast tumors sampled at the time of surgical resection without prior neoadjuvant treatment and were therefore annotated with survival data, IMS, IBS, IDS and ICR status ( Hendrickx *et al.*, 2017; Miller *et al.*, 2016). 281 of the cases did not fulfill these criteria and were therefore not annotated. Of note, 115 cases of original meta-cohort used Nagalla’s study (n=1954) were not shared within GEO, but shared within other platforms (caArray and ArrayExpress). For this reason, these samples were not included in our GXB collection ( Figure 2).

The datasets that comprise our collection are listed in Table 1 and can be searched interactively in GXB. All GEO datasets consist of unique cases with the exception for 36 cases from NUH Singapore, which are both present in the Bordet Radcliff NUH (GSE45255) dataset and the Uppsala and Singapore (GSE4922) dataset.

**Table 1.** List of datasets uploaded to GXB.

Dataset	Platforms	Diseases	Number of samples	GEO ID	References
**Bordet Radcliffe NUH dataset -** **GSE45255.GPL96**	Affymetrix Human Genome U133A Array	Mixed Breast Cancer Types	139	GSE45255	**( Nagalla *et al.*, 2013)**
**Erasmus Medical Center (EMC)** **dataset 1 - GSE2034.GPL96**	Affymetrix Human Genome U133A Array	Lymph Node Negative Breast Cancer	286	GSE2034	**( Wang *et al.*, 2005)**
**Erasmus Medical Center (EMC)** **dataset 2 - GSE5327.GPL96**	Affymetrix Human Genome U133A Array	Lymph Node Negative Breast Cancer	58	GSE5327	**( Minn *et al.*, 2007)**
**Europe and Cleveland (EMCT)** **dataset - GSE12093.GPL96**	Affymetrix Human Genome U133A Array	ER + Breast Cancer	136	GSE12093	**( Zhang *et al.*, 2009)**
**Guy's hospital dataset (GUYT2) -** **GSE9195.GPL570.fCEL**	Affymetrix Human Genome U133 Plus 2.0 Array	ER+ Breast Cancer	77	GSE9195	**( Loi *et al.*, 2008)**
**Johannes Gutenberg University** **(MAINZ) dataset - GSE11121.GPL96**	Affymetrix Human Genome U133A Array	LN- Breast Cancer	200	GSE11121	**( Schmidt *et al.*, 2008)**
**Karolinska (STO) dataset - +GPL97**	Affymetrix Human Genome U133A Array & Affymetrix Human Genome U133B Array	Mixed Breast Cancer Types	159	GSE1456	**( Pawitan *et al.*, 2005)**
**Memorial Sloan-Kettering Cancer** **Center (MSKCC) dataset -** **GSE2603.GPL96_Clinical samples**	Affymetrix Human Genome U133A Array	Mixed Breast Cancer Types	99	GSE2603	**( Minn *et al.*, 2005)**
**Nagalla 2013 reconstituted public** **dataset**	Affymetrix Human Genome U133A Array & Affymetrix Human Genome U133A2 Array & Affymetrix Human Genome U133 Plus 2.0 Array	Mixed Breast Cancer Types	1839	multiple	**( Nagalla *et al.*, 2013)**
**Guys hospital (GUYT) dataset -** **GSE6532.GPL570**	Affymetrix Human Genome U133 Plus 2.0 Array	ER+ Breast Cancer	87	GSE6532	**( Loi *et al.*, 2007)**
**John Radcliff Hospital (OXFU, OXFT)** **dataset - GSE6532.GPL96 +GPL97**	Affymetrix Human Genome U133A & U133B Array	ER+ Breast Cancer	327	GSE6532	**( Loi *et al.*, 2007)**
**TRANSBIG (TBIG) dataset -** **GSE7390.GPL96**	Affymetrix Human Genome U133A Array	Lymph Node Negative Breast Cancer	198	GSE7390	**( Desmedt *et al.*, 2007)**
**University of California San** **Francisco (YAU) dataset -** **GSE7378.GPL4685**	Affymetrix GeneChip HT-HG_U133A Early Access Array	ER+ Breast Cancer	54	GSE7378	**( Zhou *et al.*, 2007)**
**Uppsala and Singapore dataset -** **GSE4922.GPL96 +GPL97**	Affymetrix Human Genome U133A & U133B Array	Mixed Breast Cancer Types	289	GSE4922	**( Ivshina *et al.*, 2006)**

Data of the 1839 GEO-cases annotated with survival data that were previously combined and used in the Nagalla study, have been uploaded to GXB in the dataset “ Nagalla 2013 reconstituted public dataset”.

### Dataset upload into GXB

All datasets were downloaded from NCBI GEO in SOFT file format and were uploaded into GXB with the exception of the Guy's hospital dataset (GUYT2; GSE9195). Expression data in the SOFT file of this dataset was expressed as fold change. Therefore, we had to revert to reprocessing of the CEL files found attached to the GSE on GEO. In this case, the cell files were read into R (v3.2.2) using the ‘affy’ package (v1.50.0). Data was normalized using the RMA (Robust multichip averaging) and gene annotation data was added using the hgu133plus2.db package (v3.2.3).

GSE records containing data generated with different or multiple platforms have been split by platform using the import process of GXB. GSEs containing data from both clinical as *in vitro* origin (GSE2603) have been split manually using the GXB Graphical interface.

Metadata of the different studies was added to GXB both from the descriptive information found on GEO or from the method sections of the publications linked to these datasets. Short links to PMID (Pubmed) and GEO records were added.

### Construction of the Nagalla’s dataset

The constitution of the complete cohort has previously been described by ( Nagalla *et al.*, 2013). The dataset “Nagalla 2013 reconstituted public dataset” available in GXB contains only the samples that were publicly available via GEO. Briefly, raw data (CEL files) were extracted from GEO. The array platforms employed for these 13 datasets were Affymetrix U133A, U133A2, and U133 PLUS 2.0 gene chips; the 22,268 probe sets that are present in all platforms were included in the gene expression file. Data were MAS5.0 normalized using the justMAS function in the simpleaffy library from Bioconductor ( Gentleman *et al.*, 2004) using a trimmed mean target intensity of 600 without background correction. COMBAT empirical Bayes method was used to correct for batch effects ( Johnson *et al.*, 2007).

### Clinical data annotation

Gene expression data is accompanied with clinical data in CSV file format. Gene expression data and clinical data are coupled to the sample via variable “Sample ID”. We annotated a total of 1839 cases with 10-year survival (time and event), IBS (IBE, IBD), IDS (PID, WID and FID) ( Miller *et al.*, 2016) and ICR (ICR1, ICR2, ICR3 and ICR4) immune classifications ( Hendrickx *et al.*, 2017) ( Figure 2, Table 2). IMS (i.e., Basal-like, HER2-enriched, Luminal A and Luminal B) were defined using the Single Sample Predictor (SSP) algorithm by Hu ( Hu *et al.*, 2006) utilized by ( Fan *et al.*, 2006). Claudin-low tumors were identified using the method of ( Prat *et al.*, 2010). Of the 1839 samples, 251 samples were “Normal-like” in IMS classification. Therefore, these samples are not classified according to IDS. For the separate dataset containing samples of *in vitro* origin (GSE2603), survival annotations and immune classifications are not applicable. A final 281 cases were not annotated and non-classified, since for these cases either samples were not taken at the time of surgical resection, were neoadjuvant-treated or cases were not annotated with distant metastasis free survival (DMFS) time and event.

**Table 2.** Tumor classifications applied to breast cancer datasets.

Classifications	Categories	Reference
Intrinsic Molecular Subtype (IMS)	Normal-like (Normal); HER2-enriched (Her2); Basal-like (Basal); Luminal A (LumA); Luminal B (LumB); Claudin low (ClaudinLow)	( Hu *et al.*, 2006)
Immune Benefit Status (IBS)	Immune Benefit-Enabled (IBE); Immune Benefit-Disabled (IBD)	( Miller *et al.*, 2016; Nagalla *et al.*, 2013)
Immunogenic Disposition Status (IDS)	Poor Immunogenic Disposition (PID); Weak Immunogenic Disposition (WID); Favorable Immunogenic Disposition (FID)	( Miller *et al.*, 2016; Nagalla *et al.*, 2013)
Immunologic Constant of Rejection (ICR)	ICR1; ICR2; ICR3; ICR4	( Galon *et al.*, 2013; Hendrickx *et al.*, 2017)

To enable comparisons between datasets and to facilitate efficient data analysis, the clinical data was harmonized to reflect a nomenclature similar to that of The Cancer Genome Atlas (TCGA). Clinical variable names and availability in datasets are listed in Table 3. In general, variable values have been replaced by descriptive values (e.g. “1” and “0” are replaced by “ER+” and “ER-”, respectively). For disease free survival, variable values have been adapted to “DiseaseFree” or “Recurred/Progressed”, and for distant metastasis survival to “DistantMetastasisFree” and “DistantMetastasis”. Numeric values of variable “tumor size” have been converted to units in cm for all datasets. This variable was used to generate the additional variable pathology T stage according to the 7th edition of the AJCC staging system for breast cancer ( Edge & Compton, 2010). For tumors with a diameter larger than 5 cm, pathology T stage could be either T3 or T4, therefore value “T3/T4” has been assigned to these cases.

**Table 3.** Clinical data availability.

Clinical variable	Available in *N* datasets
IMS	13
IBS	13
IDS	13
ICR	13
DMFS 10Y EVENT	13
DMFS 10Y TIME	13
Disease free survival event	11
Disease free survival time	11
Distant metastasis free survival event	6
Distant metastasis free survival time	6
Age at initial pathologic diagnosis	8
Lymph node status	8
ER status	8
PR status	5
Histology differentiation grade	7
Tumor size	8
Pathology T Stage	8
Type treatment, bone metastasis event, bone metastasis free survival time, breast cancer cause of death, HER2 status, histologic diagnosis, lung metastasis event, lung metastasis free survival time, lung metastasis gene expression signature status, vital status, angio invasion indicator, disease specific survival time, genetic grade signature status sws classifier, GGI indicator, lymph nodes examined count, number of lymph nodes positive, lymphocyte infiltration, molecular subtype, NPI, overall survival, p53 mutation status, probability by sws classifier, RFS 5Y EVENT, risk AOL indicator, risk NPI indicator, risk SG, risk veridex indicator, tissue type, van 't Veer signature.	<2

Standardized clinical datasets can be found in the ‘downloads’ tab in GXB under the heading “additional files”. All datasets start with the following 21 clinical variables in fixed order: "sample.ID", "array sample id", "sample title", "series", "IMS", "IBS", "IDS", "ICR", "DMFS_10Y_EVENT", "DMFS_10Y_TIME", "disease free survival event", "disease free survival years", "distant met free survival event", "distant met free survival", "age at initial pathologic diagnosis", "lymph node status", "ER status", "PR status", "histology differentiation grade", "tumor size cm", and "pathology T stage". In case one of these variables is not available in a specific dataset, values in this column are all NA.

Group sets for IBS/IDS, ICR cluster, Lymph Node (LN) Status, IMS, Histological grade, stage and Estrogen Receptor (ER) status were defined with matching differential gene expression rank lists. Rank lists are based on differential gene expression between two relevant groups for each group set: IBD-FID *vs* IBE-FID (IBS/IDS); ICR1 *vs* ICR4 (ICR1/ICR4); LN+ *vs* LN- (LN status); G1 *vs* G3 (histological grade); ER+ *vs* ER- (ER status). For IMS, no rank list was generated, as this variable is not ordered. For tumor stage, no rank list was generated because the spread of samples between categories was small (R scripts for this harmonistaion have been made available on Github.).

## Dataset demonstration

### Utilization of GXB

The GXB software has been described in detail in a recent publication ( Speake *et al.*, 2015). This custom software interface provides users with a means to easily navigate and filter the dataset collection available at http://breastcancer.gxbsidra.org/dm3/landing.gsp. A web tutorial is also available online: http://breastcancer.gxbsidra.org/dm3/tutorials.gsp#gxbtut.

### Example case: Expression of HLA-G across ICR groups

In GXB, users can search interactively for a specific gene of interest. Differential expression across different group sets can be observed in the graphical interface, either in bar or box plots. For illustrative purposes, we choose to evaluate the abundance of the HLA-G transcripts across ICR groups.

HLA-G is a non-classical class I gene of human Major Histocompatility Complex that is primarily expressed on fetal derived placental cells ( Ellis *et al.*, 1990). In contrast to its classical counterparts, HLA-G does not initiate immune responses, but instead has immunosuppressive effects ( Naji *et al.*, 2014; Rouas-Freiss *et al.*, 1997). Expression of HLA-G has been reported in a variety of cancers, including breast cancer, and has been assigned a role in tumor immune escape ( Naji *et al.*, 2014; Rouas-Freiss *et al.*, 1997; Swets *et al.*, 2016; Zeestraten *et al.*, 2014).

Concerning its role in tumor immunity, it may be of interest to investigate whether HLA-G expression is elevated in breast tumors of specific immune phenotypes. The ICR gene signature segregates breast tumors into four immune phenotype groups based on the expression of genes underlying immune-mediated tissue-specific destruction, with ICR1 having the lowest and ICR4 the highest expression of this signature ( Bedognetti D *et al.*, in press).

**Figure 3.**
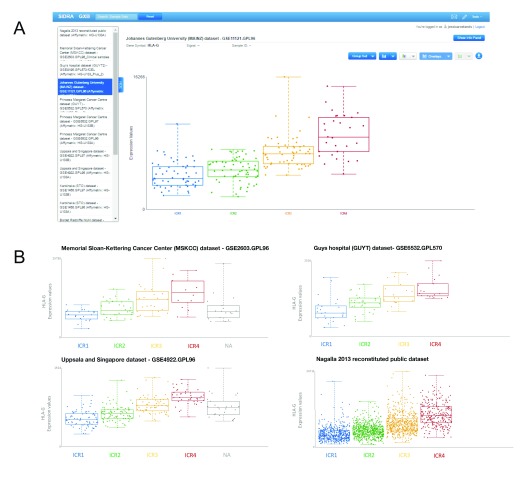
Illustrative example of abundance of HLA-G transcripts across ICR groups in multiple breast cancer datasets in GXB. ( **A**) Cross Project View in GXB showing HLA-G expression across ICR groups. ICR represents the immune gene signatures observed in association with tissue-specific destruction. In this view of GXB, expression of HLA-G can be visualized across projects listed on the left. ( **B**) Boxplots of HLA-G expression across ICR groups of three additional representative datasets selected from the dataset collection and the complete dataset including all annotated cases (right bottom plot). Plots indicate an increased HLA-G expression in breast tumors with a high expression of ICR genes. ICR, Immunologic Constant of Rejection.

To compare HLA-G expression across ICR groups using the breast cancer datasets uploaded to GXB, we start by selecting a dataset. Users can decide to first explore the Nagalla 2013 dataset containing the total of 1839 annotated cases from all uploaded datasets to define trends to subsequently check their consistency over the different datasets. Alternatively, users can first explore the individual datasets. After opening one of the datasets: 1) the gene of interest, HLA-G, can be identified using the search box in the upper left corner of the user interface. Upon selection of “HLA-G” in the left panel, the central panel displays the expression values of this gene for all samples as a bar chart. For some of the transcripts, as is the case for the HLA-G transcript, multiple probes are available. Probe ID can be displayed by clicking “Tools” in the central panel and clicking “Show Probe ID” in the dropdown list. In this example, which is for illustrative purposes only, we selected probe ID 211528_X_AT at random. 2) Sample grouping is default as “All sample”, it is changed by selecting “Immunologic Constant of Rejection” and 3) plot type is set to “Box Plot” in drop down menus in the central panel. The central panel now presents a graphical display of the observed abundance of HLA-G transcripts in breast cancer samples across the different ICR groups, each sample is represented by a single point in a boxplot ( Figure 3A). A tendency of increased HLA-G expression in groups with the highest expression of ICR genes can be observed. 4) To verify whether this trend can also be observed in other breast cancer datasets, GXB’s “Cross Project View” is used. By selecting “Cross Project View” in the “Tools” drop-down menu located in the top right corner of the user interface, a list of available datasets/projects appears in the left pane. By consecutive selection of single datasets, box plots with HLA-G transcripts across ICR groups are displayed for each individual dataset.

Each of the boxplots corresponding to the 13 datasets show a similar pattern, indicating an increased HLA-G expression in breast tumors with a high expression of ICR genes (representative plots are shown in Figure 3B). In the combined dataset containing the total of 1839 annotated cases from these datasets, this trend is also observed ( Figure 3B). From a biological perspective, increased expression of an immunosuppressant in an immunologically active tumor would be in line with our current view of the tumor microenvironment. Pro-inflammatory tumor environments, as observed in ICR4 tumors, also show counter regulatory mechanisms to suppress the immune system ( Bedognetti *et al.*, 2015; Galon *et al.*, 2013).

This observation made by exploring transcriptome data in GXB provides an interesting starting point for further analysis. Statistical analysis of this potential association is required and, of course, the clinical relevance of the observed difference in abundance of transcripts should be determined. Most importantly, the functional relevance of HLA-G expression depends on its interaction with inhibitory receptors including ILT2, ILT4 and KIR2DL4 ( LeMaoult *et al.*, 2005). Therefore, combined analysis of both HLA-G and these inhibitory receptors is suggested in future analyses.

This example illustrates the convenience of exploring gene expression data in GXB. The browser facilitates intuitive navigation and visualization of gene expression across different group sets.

### Differential gene expression between IBS/IDS subgroups

The breast cancer datasets uploaded in GXB are provided with a rich context of immune classifications and clinical parameters. As opposed to start a search with a specific gene of interest, as presented in the HLA-G example case, differential gene expression between *groups* of interest can be explored in GXB by evaluation of gene rank lists. Here, we demonstrate the use of GXB to explore differential gene expression across IBS/IDS groups.

The IDS group set is based on an immune metagene model segregating breast tumors in groups of different immunogenic dispositions: PID, WID and FID ( Nagalla *et al.*, 2013). The prognostic value of this classification is dependent on the molecular subtype and the proliferative capacity of the tumor, hereby segregating tumors in IBE and IBD groups, with and without prognostic value of the IDS, respectively. Since the hypothesis is that IBE-FID tumors confer metastasis-protective potential and IBD-FID tumors do not, transcriptional differences between these specific subgroups are of particular interest and have systematically been analyzed by Miller *et al.* (2016).

**Figure 4.**
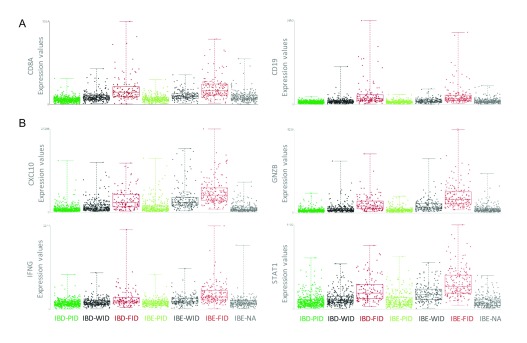
GXB overview of expression of genes with known roles in tumor immunology across IBS/IDS subgroups in reconstituted Nagalla’s breast cancer dataset. ( **A**) Expression values of CD8 and CD19, indicators of immune cell infiltration, are similar in IBD-FID and IBE-FID groups, indicating equal immune cell infiltration in these subgroups. ( **B**) Expression values of CXCL10, GNZB, IFNG and STAT1, markers of immune functional orientation, are increased in the IBE-FID group compared with IBD-FID, indicating a differential functional orientation of the immune infiltrate between IBD-FID and IBE-FID tumors. IBE/D, Immune Benefit Enabled OR Disabled; F/P/WID, Favorable OR Poor OR Weak Immune Disposition.

The Nagalla 2013 reconstituted dataset containing all annotated cases of this GXB breast cancer instance (n=1839) is used to explore differential gene expression between IBE-FID and IBD-FID tumors in GXB. Group set “Immune Benefit Status” is selected and corresponding gene rank list “IBD-FID *vs* IBE-FID” will load in the left panel by default. Filtering for specific immune gene categories, e.g. cytokine and chemokine ligands, cytokine and chemokine receptors, B and T cell signaling, and antigen presenting cell processing, is possible by selecting gene list category in the rank list menu. Exploring the expression of genes with known roles in tumor immunology reveals two important observations: 1) markers of immune cell infiltration, including CD8, CD3, CD19 and CD2, show similar expression in IBD-FID and IBE-FID subgroups ( Figure 4A); while (2) markers of immune functional orientation, including CXCL10 (tissue rejection chemokine), GZMB (cytotoxic effector molecule), INFG and STAT1 (Th1 polarization), show differential expression across IBD-FID and IBE-FID groups ( Figure 4B). A comprehensive statistical analysis of expression of these and other immune-related genes confirmed these observations, suggesting that while the composition of the immune infiltrate is similar in these tumors, the functional molecular orientation determines the metastasis-protective phenotype ( Miller *et al.*, 2016).

This demonstration indicates that GXB allows for easy and efficient visualization of differential gene expression between subgroups. Subsequently, elaborate statistical analysis is required to confirm the differences in gene expression observed in GXB.

### Overview of breast cancer immune classifications in GXB

Since this GXB data collection is provided with multiple immune classifications of breast cancer, it is interesting to visualize the relationship between these classifications in GXB. The overlay feature in GXB can be used to visualize the assignment of different classifications to individual samples simultaneously.

**Figure 5.**
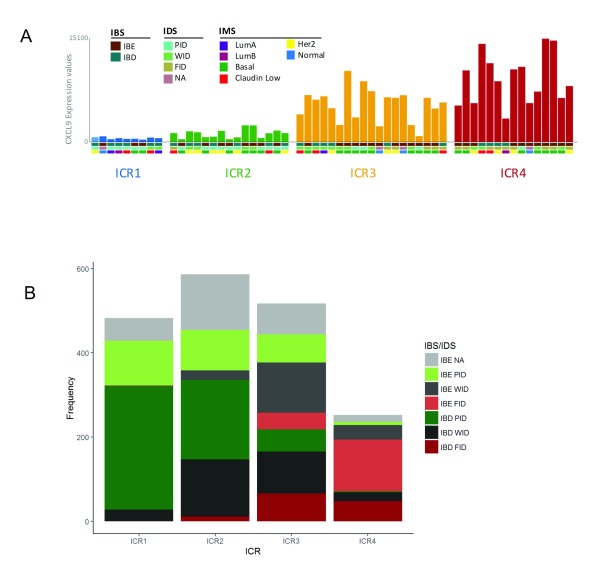
Overlay of immunologic classifications in breast cancer as evaluated in GXB. ( **A**) Bar graph showing CXCL9 expression in individual samples from Erasmus Medical Center (EMC) dataset 2 split by ICR (single bar represents single case). Overlay of additional variables IBS, IDS and IMS is shown ( http://breastcancer.gxbsidra.org/dm3/miniURL/view/Lv). ( **B**) Frequency plot showing number of breast cancer cases across IBS/IDS subgroups split by ICR cluster. ICR, Immunologic Constant of Rejection; IBE/D, Immune Benefit Enabled OR Disabled; F/P/WID, Favorable OR Poor OR Weak Immune Disposition.

To illustrate this overlay option, we choose to select the Erasmus Medical Center dataset 2 (EMC2) with CXCL9 expression, as this is one of the chemokines included in the ICR gene signature. Graphical representation in GXB is set to bar plot and group set ICR is selected. As anticipated, the CXCL9 expression gradually increases from ICR1-ICR4. The drop down menu “Overlays” is used to add multiple layers of additional variables, “IBS”, “IDS” and “IMS”. Boxes underneath the individual bars (each bar represents a single case) display the assigned classifications ( Figure 5A). When comparing IBS classifications across ICR groups, it is evident that IBE tumors are frequently assigned to the higher ICR clusters, ICR3 and ICR4, while IBD tumors tend to concentrate to the clusters with a low expression of the ICR signature (ICR1, ICR2) ( Figure 5A). This result is consistent with our previous observations: pathways that distinguish IBE and IBD are associated with the immune functional orientation of the tumor, and genes in these same pathways are crucial components of the ICR signature ( Bedognetti *et al.*, 2015; Miller *et al.*, 2016).

IDS relates to the ICR classification in a similar manner. FID tumors are mostly assigned to ICR4, while WID tumors are frequently classified to intermediate clusters (ICR2 and ICR3), and PID tumors prevail in the ICR1 cluster ( Figure 5A). This observation is also in line with our expectations, the IDS classification is based on an immune metagene model that relies on immune gene subclusters that reflect the relative abundance of tumor-infiltrating immune cells ( Nagalla *et al.*, 2013). As markers of immune cell infiltration are also included in the ICR signature, IDS is closely associated with ICR.

For a more comprehensive overview of the relationship between different immune classifications in breast cancer, the overlay of immune classifications was evaluated in the Nagalla 2013 reconstituted public dataset (n=1839). The observations made in the EMC2 dataset (n=58; Figure 5A) are also apparent in the dataset containing all annotated cases of this GXB breast cancer instance ( Figure 5B). Moreover, in this dataset it is clearly visible that IBS/IDS subgroups with an improved prognosis are more prevalent in the ICR4 cluster. For example, IBE-FID tumors are relatively more frequently assigned to ICR4 compared with IBD-FID. Vice versa, IBD-PID tumors are proportionally more frequently observed in the ICR1 cluster compared with IBE-PID tumors, which are in comparison more frequently assigned to ICR2 ICR3.

The overlay of the different immune classifications demonstrates a coherency between the IBS/IDS classification and the ICR clusters. Bearing in mind that the ICR signature is associated with a broader phenomenon of immune-mediated, tissue-specific destruction, this coherency strengthens the hypothesis of a common final pathway of tissue destruction.

## Conclusions

In this data note, we highlighted the opportunities provided by the availability of public datasets. We uploaded 13 public datasets on human breast cancer, including a combined dataset, with harmonized clinical data annotation and immune classification to GXB to facilitate the reuse of gene expression data. The use of GXB to explore gene expression and the different possible approaches were illustrated by the following: (1) an example case of a specific gene of interest, HLA-G; (2) comparison of gene expression between specific subgroups, IBD-FID vs IBE-FID; and (3) the evaluation of the relationship between different categorical variables, IBS/IDS and ICR immune classifications. To conclude, GXB provides a convenient environment to explore gene expression profiles in the context of breast cancer.

## Data availability

All datasets included in our curated collection are available publicly via the NCBI GEO website: http://www.ncbi.nlm.nih.gov/geo/, and are referenced throughout the manuscript by their GEO accession numbers (e.g. GSE7390). Signal files and sample description files can also be downloaded from the GXB tool under the “downloads” tab.
