# Supplementary material for: A collection of annotated and harmonized human breast cancer transcriptome datasets, including immunologic classification
Source: F1000Res. 2018 Feb 9;6:296. Originally published 2017 Mar 20. [Version 2] doi: 10.12688/f1000research.10960.2 (PMC5820610; doi:10.12688/f1000research.10960.2)
Supplement: Peer review history

# Peer review: Referee response for version 2

**DOI:** 10.5256/f1000research.15014.r30723

**Published:** 2018-02-19

**Authors:** Benjamin Haibe-Kains

**Affiliations:** 1 Princess Margaret Cancer Centre, University Health Network, Toronto, ON, Canada

I thank the authors for their response. Many of my suggestions have not been implemented. If these limitations are clearly stated in the paper, I think this database could benefit the community.

I have read this submission. I believe that I have an appropriate level of expertise to confirm that it is of an acceptable scientific standard.

# Peer review: Referee response for version 2

**DOI:** 10.5256/f1000research.15014.r30722

**Published:** 2018-02-09

**Authors:** Christos Hatzis

**Affiliations:** 1 Breast Medical Oncology, Yale School of Medicine, New Haven, CT, USA

My concerns/queries have been addressed in the revised version.

I have read this submission. I believe that I have an appropriate level of expertise to confirm that it is of an acceptable scientific standard.

# Peer review: Referee response for version 1

**DOI:** 10.5256/f1000research.11813.r24824

**Published:** 2017-09-26

**Authors:** Christos Hatzis

**Affiliations:** 1 Breast Medical Oncology, Yale School of Medicine, New Haven, CT, USA

Roelands *et al* present a web application that provides real-time analysis and visualization of a large compendium of microarray datasets generated from primary breast cancer biopsies. The uniqueness and distinct utility of this dataset is the focus on immune-related signatures. Before discussing potential shortcomings according to this reviewer's opinion, the authors should be commended for investing the energy and resources to develop and implement a focused public repository that would facilitate further analyses.

 Although the overall objective behind this website and tools is laudable, I believe that the approach followed, as presented in the manuscript, has several potential shortcomings that would be worth addressing.

1. Regarding the protocols followed, I would echo the comments by the first reviewer and question the use of MAS5 and COMBAT, given that more robust algorithms are now available. This may be a key issue given the quantitative nature of many of the indices used. Also, it is not clear that this approach removes bias between the older Affymetrix platforms and the newer ones (PLUS2.0). On page 5 it is mentioned that "the 22,268 probe sets present in each of these platforms were included", but the PLUS 2.0  chips include twice as many. They probably meant to say "present in all platforms".
2. In terms of whether sufficient details were provided to allow replication, it would be great if the authors provided the complete set of scripts used to pre-process and normalize the data included in the analysis. This will allow transparent assessment of the methods and replication of the dataset if needed. 
3. Related to the type of data included, I believe that the resource would be more valuable if additional clinically-relevant information was provided with each sample. For example, disease progression clearly depends on treatment and treatment information is missing entirely - we do not know whether patients even received any treatment at all. It would be useful to curate treatment information available in the original datasets. Also, it may be worth providing annotations for additional immune signatures (e.g. Rody *et al*, 2011 ^1^) and also of additional breast cancer subtypes (e.g. Lehmann *et al*,  2016 ^2^). This will allow a more thorough assessment of the overlap between the various signature-defined subtypes. Finally, although there are not as many profiles from breast cancer metastasis available in GEO, it may be worth considering curating those and including them in the platform. It would be extremely interesting to know how the immune signatures differ between primary and metastatic samples, and whether the trends suggest immune evasion.
4. Finally, from a usability standpoint, I have a few suggestions outlined below:
  - Although the functionality may be available, I found it very difficult to filter samples based on a particular feature. For example, I was trying to do the HLA-G by ICR group analysis only for the ER-negative or basal cases, but could not find an easy way to do that.
  - It would be useful to provide tools for correlational analysis of two or more genes (e.g. co-expression patterns) and heat maps. The one gene at a time visualization is very restrictive.

Minor points:

1. Worth considering having another table after Table 1 that summarizes the immune subtype signatures and annotations with references. Intrinsic subtype signatures used can also be summarized in that table. That will also eliminate the need to explain the acronyms (e.g. Table 2 is marred by too many acronyms IMS, IBC, IDS, ICR that are not explained).
2. When running the HLA-G example as outlined in the manuscript, I was presented with 4 different HLA-G transcripts with no explanation as to what was the difference between them. Are these different probe sets? If so, that should probably be explained. Also, in that case, is there a recommendation as to which of these probe sets is the most representative (e.g. based on specificity, range, variance etc).

I have read this submission. I believe that I have an appropriate level of expertise to confirm that it is of an acceptable scientific standard, however I have significant reservations, as outlined above.

## References

[1] RodyAKarnTLiedtkeCPusztaiLRuckhaeberleEHankerLGaetjeRSolbachCAhrAMetzlerDSchmidtMMüllerVHoltrichUKaufmannM: A clinically relevant gene signature in triple negative and basal-like breast cancer. *Breast Cancer Res*.2011;13(5) : 10.1186/bcr3035 R97 10.1186/bcr3035 21978456PMC3262210

[2] LehmannBDJovanovićBChenXEstradaMVJohnsonKNShyrYMosesHLSandersMEPietenpolJA: Refinement of Triple-Negative Breast Cancer Molecular Subtypes: Implications for Neoadjuvant Chemotherapy Selection. *PLoS One*.2016;11(6) : 10.1371/journal.pone.0157368 e0157368 10.1371/journal.pone.0157368 27310713PMC4911051

# Response

**Published:** 2018-01-21

**Authors:** Wouter Hendrickx

**Affiliations:** Sidra Medical and Research Center, Qatar

Major points:

- Regarding the protocols followed, I would echo the comments by the first reviewer and question the use of MAS5 and COMBAT, given that more robust algorithms are now available. This may be a key issue given the quantitative nature of many of the indices used. Also, it is not clear that this approach removes bias between the older Affymetrix platforms and the newer ones (PLUS2.0). On page 5 it is mentioned that "the 22,268 probe sets present in each of these platforms were included", but the PLUS 2.0 chips include twice as many. They probably meant to say "present in all platforms".

***The primary goal of this data note was to share the transcriptomic and annotation data associated with this previous publication (in GXB defined as “the Nagalla 2013 reconstituted public dataset”) and the breast cancer transcriptomic datasets from GEO that it constitutes of in a more interactive, comprehensible format to facilitate usage of the data and did not involve data processing. The selection of 22,268 probe sets, combination and normalization of the 13 datasets was also previously performed (Nagalla et al).*** ***For your second point, we indeed meant the probe sets that are present in all platforms, for this reasons probe sets that are exclusively present in Affymetrix U133 PLUS2.0 are not included in these 22,268 probe sets. We have revised this sentence in the manuscript accordingly.***

- In terms of whether sufficient details were provided to allow replication, it would be great if the authors provided the complete set of scripts used to pre-process and normalize the data included in the analysis. This will allow transparent assessment of the methods and replication of the dataset if needed. 

***The uploads from individual GEO datasets into GXB did not involve any data processing. For the Nagalla et al this has been performed as part of the work described in Nagalla et al. This data note did involve clinical data harmonization performed in R, scripts have been made available on*** ***Github*** ***.***

- Related to the type of data included, I believe that the resource would be more valuable if additional clinically-relevant information was provided with each sample. For example, disease progression clearly depends on treatment and treatment information is missing entirely - we do not know whether patients even received any treatment at all. It would be useful to curate treatment information available in the original datasets. Also, it may be worth providing annotations for additional immune signatures (e.g. Rody *et al*, 2011 ^1^) and also of additional breast cancer subtypes (e.g. Lehmann *et al*,  2016 ^2^). This will allow a more thorough assessment of the overlap between the various signature-defined subtypes. Finally, although there are not as many profiles from breast cancer metastasis available in GEO, it may be worth considering curating those and including them in the platform. It would be extremely interesting to know how the immune signatures differ between primary and metastatic samples, and whether the trends suggest immune evasion.

 

***When uploading these GEO datasets into GXB, all available clinical data on GEO was also transferred to GXB. For some of the datasets (e.g. GSE4922), a treatment parameter is available which can be selected as a parameter using the overlay function in the bar plot generated in GXB. We completely agree that information on adjuvant treatment is essential for interpretation of the supplied survival data (Distant Metastasis Free Survival and Disease Free Survival). For proper survival analysis, we recommend users to download the csv file supplied which can be found under the “Downloads”-tab of each dataset and use available treatment information for stratification.***

***Addition of more gene signatures can indeed be valuable to compare immune based classifications as demonstrated in this data note for Immunological Constant of Rejection (ICR) classification and Immune Benefit Status (IBS)/ Immune Disposition Status (IDS). Similarly, uploading additional GEO datasets that contain matched primary and metastatic samples would be very interesting and allow for comparison of both immune microenvironments. Although not in the scope of the current data note, we will definitely take these suggestions into consideration for further development of the GXB breast cancer instance.***

 

- Finally, from a usability standpoint, I have a few suggestions outlined below:

-Although the functionality may be available, I found it very difficult to filter samples based on a particular feature. For example, I was trying to do the HLA-G by ICR group analysis only for the ER-negative or basal cases, but could not find an easy way to do that.

***It is indeed possible to visualize the expression of a gene of interest, for example HLA-G, in a specific subgroup of samples like all ER-negative or basal cases. The most convenient way to do this is to (1) select barplot, (2) set group by Molecular Subtype and (3) overlay the barplots with ICR group annotation. To make the figure easier to analyze, you can subsequently sort by ICR.***

- It would be useful to provide tools for correlational analysis of two or more genes (e.g. co-expression patterns) and heat maps. The one gene at a time visualization is very restrictive.

***We realize that gene per gene visualization has its limitations and analysis of groups coordinately expressed genes provides more biological significance. For this reason, the GXB development team has already created a*** ***module analysis tool (MAT)*** ***that*** ***analyses expression data to find pre-defined groupings of co-expressed genes (modules) to obtain a molecular fingerprint of gene expression for each individual sample in your dataset*** ***.*** ***MAT has already been applied to many datasets with samples of various immune related diseases and we are considering to also use this platform for cancer transcriptomic datasets. As the desired number of group comparisons in breast cancer (i.e., Molecular Subtypes, Immunologic classifications, Pathology T Stage) is higher compared to the existing group comparisons, some work is required before we can implement these dataset in this tool.***

Minor points:

- Worth considering having another table after Table 1 that summarizes the immune subtype signatures and annotations with references. Intrinsic subtype signatures used can also be summarized in that table. That will also eliminate the need to explain the acronyms (e.g. Table 2 is marred by too many acronyms IMS, IBC, IDS, ICR that are not explained).

***We have added the suggested table to the manuscript. This is both useful when reading the manuscript as well as for the use of this breast cancer GXB application, thank you for this suggestion.***

- When running the HLA-G example as outlined in the manuscript, I was presented with 4 different HLA-G transcripts with no explanation as to what was the difference between them. Are these different probe sets? If so, that should probably be explained. Also, in that case, is there a recommendation as to which of these probe sets is the most representative (e.g. based on specificity, range, variance etc).

***These different HLA-G transcripts are indeed different probe sets. If you are interested to find and select a specific probe ID, you can select “Show Probe ID” under “Tools”. This GXB data portal does not give an explanation on the difference between these probes. For this information we would like to refer to the Affymetrix website*** ***.  For the HLA-G example, we just took a single probe at random: 211528_X_AT. We added this information in version 2 of the manuscript.***

# Peer review: Referee response for version 1

**DOI:** 10.5256/f1000research.11813.r24330

**Published:** 2017-07-24

**Authors:** Benjamin Haibe-Kains

**Affiliations:** 1 Princess Margaret Cancer Centre, University Health Network, Toronto, ON, Canada

In this manuscript, Roelands *et al.* describe the implementation of the gene expression browser (GXB), a database and web-application integrating 13 breast cancer datasets containing gene expression and clinical data for a total of almost 2000 patients. The authors focused their case studies on the investigation of immune-related genes and subtypes and made their curated data publicly available. I think GXB is a welcome contribution to the breast cancer research community and its public availability will help other scientists to leverage this valuable collection of datasets. However, I have several concerns that need to be addressed before indexing, as listed below.

 The manuscript is well written and easy to understand. 

**Major comments**

 In Table 1 and on the front page of the web-application, Princess Margaret Cancer Centre is listed in the sample set name even though these data have not been generated in this institution, which is misleading.

 When selecting "Breast Cancer", which I assumed contained the whole database, many datasets got filtered out, which is confusing.

 I tried AURKA and selected GSE9195 as dataset. Then I played a bit with the barplot to overlay different kinds of information. I added DMFS 10Y (categorical) and sorted the patients based on this but there were some patients with very low DMFS at the beginning and the end of the plot, which is confusing.

 Since the authors are dealing with survival data, I was expecting survival statistics and/or survival curves but I did not see any in GXB or the manuscript. Figure 1 is somehow misleading as these plots do not seem to be part of the web-application.

 The authors decided to limit their database to Affymetrix data. In that case, I suggest the authors reprocess all the CEL files consistently using (f)RMA and up-to-date chip description files (CDF), such as BrainArray CDFs. This would increase the consistency across all the datasets.

 Given the authors' large collection of datasets, mining each dataset separately is cumbersome. Implementing meta-analysis pipelines would help draw an overall conclusion before digging into dataset-specific results (e.g., Figure 3). This is what the authors seem to have done with the "Nagalla" dataset that is a compendium of all the datasets reprocessed using MAS5 and further corrected with ComBat. It is not clear why the authors used MAS% to normalize the data in this case vs (f)RMA for some other datasets. Please clarify. Why not recommending users to first explore Nagallia before going after each dataset separately to detect trends that may be (in)consistent with the majority of the datasets?

 There have been many molecular subtyping schemes published, including the Integrative Subtypes (IntClust; Curtis et al, Nature 2012), the Subtype Classification Models (SCMGENE, SCMOD1, SCMOD2; Haibe-Kains et al, JNCI 2012) and the Absolute Assignment of Breast Cancer Intrinsic Molecular Subtype (AIMS; Paquet et al, JNCI 2015). The authors should discuss the rationale for their choice of relying solely on PAM50.

**Minor comments**

 Table 2 would be better represented as a barplot or a similar figure.

 GXB would benefit from the inclusion of normal samples (from healthy patients or adjacent normal samples). METABRIC, TCGA and GTEx are relevant data sources for such gene expression profiles.

I have read this submission. I believe that I have an appropriate level of expertise to confirm that it is of an acceptable scientific standard, however I have significant reservations, as outlined above.

# Response

**Published:** 2017-09-17

**Authors:** Wouter Hendrickx

**Affiliations:** Sidra Medical and Research Center, Qatar

***Answers are in Italic and bold***

****

**Major comments**

 In Table 1 and on the front page of the web-application, Princess Margaret Cancer Centre is listed in the sample set name even though these data have not been generated in this institution, which is misleading.

***Names of datasets have been changed from “Princess Margaret Cancer Centre dataset - GSE6532.GPL96” and “Princess Margaret Cancer Centre dataset - GSE6532.GPL97”, to “John Radcliff Hospital (OXFU, OXFT) dataset- GSE6532.GPL96” and “John Radcliff Hospital (OXFU, OXFT) dataset- GSE6532.GPL97” respectively. “Princess Margaret Cancer dataset- GSE6532.GPL570” was changed to “Guys hospital (GUYT) dataset- GSE6532.GPL570”. Tables and figures throughout the manuscript have been revised accordingly.***

 When selecting "Breast Cancer", which I assumed contained the whole database, many datasets got filtered out, which is confusing.

***Thank you for noticing this. Only a single disease type can be assigned per dataset. We can change ER+ and LN- datasets to disease type: Breast Cancer , effectively disabling the user to filter datasets based on ER+ and LN status. We preferred changing the name of Breast cancer (general) to "mixed breast cancer types" for clarity.***

 I tried AURKA and selected GSE9195 as dataset. Then I played a bit with the barplot to overlay different kinds of information. I added DMFS 10Y (categorical) and sorted the patients based on this but there were some patients with very low DMFS at the beginning and the end of the plot, which is confusing.

***We were able to reproduce the barplot you described. By sorting by the categorical variable DMFS 10Y EVENT, you only sort based on this variable (DistantMetastasisFree or DistantMetastasis). The continuous counterpart of this variable (DMFS 10Y TIME) is not taken into account when you perform the sorting, explaining your observation. An alternative is to sort based on the continuous variable DMFS 10Y TIME, though this sorting results in a plot that mixes DistantMetastasisFree and DistantMetastasis categories. Unfortunately, subsequent sorting based on 2 variables is not possible using GXB. We have suggested this improvement to the GXB developing team.***

 Since the authors are dealing with survival data, I was expecting survival statistics and/or survival curves but I did not see any in GXB or the manuscript. Figure 1 is somehow misleading as these plots do not seem to be part of the web-application.

***Survival statistics are not yet part of the GXB web-application. To prevent any misconception, we have added an extra sentence in the legend of figure 1: “This figure is for explanatory purposes only and does not serve as a demonstration of the GXB web application.”***

 The authors decided to limit their database to Affymetrix data. In that case, I suggest the authors reprocess all the CEL files consistently using (f)RMA and up-to-date chip description files (CDF), such as BrainArray CDFs. This would increase the consistency across all the datasets.

***The purpose of this data note is to share existing data from GEO in a more comprehensible format and does not involve any data processing. We had to make an exception for dataset GSE9195.GPL570, since only raw format was*** ***available on GEO.***

 Given the authors' large collection of datasets, mining each dataset separately is cumbersome. Implementing meta-analysis pipelines would help draw an overall conclusion before digging into dataset-specific results (e.g., Figure 3). This is what the authors seem to have done with the "Nagalla" dataset that is a compendium of all the datasets reprocessed using MAS5 and further corrected with ComBat. It is not clear why the authors used MAS5 to normalize the data in this case vs (f)RMA for some other datasets. Please clarify.

***Normalization of the complete Nagalla cohort was previously performed as described in Nagalla et al. 2013 and Miller et al. 2016 To make this data available as published in this article, we used the same data file to upload to GXB.*** ***Normalization is different between datasets because the data was transferred straight from GEO and different contributors use different methods depending on the times it was performed and the need of their specific work.***

 Why not recommending users to first explore Nagallia before going after each dataset separately to detect trends that may be (in)consistent with the majority of the datasets?

***In this dataset collection, it is indeed possible to first explore the Nagalla dataset, which is a dataset that includes all samples from the collection. This advantage is specific for this breast cancer compendium of datasets, so your suggestion is a very good strategy to explore these datasets. We have added this recommendation in the text of the Dataset Demonstration section of the data note.***

 There have been many molecular subtyping schemes published, including the Integrative Subtypes (IntClust; Curtis et al, Nature 2012), the Subtype Classification Models (SCMGENE, SCMOD1, SCMOD2; Haibe-Kains et al, JNCI 2012) and the Absolute Assignment of Breast Cancer Intrinsic Molecular Subtype (AIMS; Paquet et al, JNCI 2015). The authors should discuss the rationale for their choice of relying solely on PAM50.

***Categorization using the PAM50 molecular subtyping was performed as part of the research performed by Nagalla et al. 2013 and Miller et al. 2016. These annotated datasets were uploaded in GXB as described in this data note. The choice to rely on this 50-gene classifier was dependent on its high prognostic and predictive value of this subtyping method in combination with the high clinical applicability of PAM50 testing.***

**Minor comments**

 Table 2 would be better represented as a barplot or a similar figure.

***For the purpose of listing which clinical variables are available in how many datasets from the collection, we found a table the more appropriate option.***

 GXB would benefit from the inclusion of normal samples (from healthy patients or adjacent normal samples). METABRIC, TCGA and GTEx are relevant data sources for such gene expression profiles.

***Comparing healthy control tissue with cancerous tissue on gene expression level indeed represents an interesting aspect that can be explored using the GXB browser. At this moment, different types of dataset collections have already been uploaded to GXB, including case versus control comparisons. For example, a dataset with gene expression of whole blood samples from both*** ***lung cancer patients and healthy controls*** ***and a dataset with*** ***head and neck cancer and cervical cancer tissue samples, matched with site-matched normal epithelial samples*** ***are currently available. The TCGA, METABRIC dataset and datasets in the GTEx portal are valuable resources that can be used to upload new datasets into GXB. We will definitely take this suggestion into consideration for our new dataset collection to upload into GXB.***

***Discussed changes will be applied in version 2 of the manuscript.***
